# The Characterization of R2R3-MYB Genes in *Ammopiptanthus nanus* Uncovers That the miR858-AnaMYB87 Module Mediates the Accumulation of Anthocyanin under Osmotic Stress

**DOI:** 10.3390/biom13121721

**Published:** 2023-11-29

**Authors:** Batu Sumbur, Fei Gao, Qi Liu, Dandan Feng, Jie Bing, Tashi Dorjee, Xuting Li, Huigai Sun, Yijun Zhou

**Affiliations:** 1 Key Laboratory of Mass Spectrometry Imaging and Metabolomics, Minzu University of China, National Ethnic Affairs Commission, Beijing 100081, China; songbuerbatu@muc.edu.cn (B.S.); gaofei@muc.edu.cn (F.G.); 20400256@muc.edu.cn (Q.L.); 21040062@muc.edu.cn (D.F.); 20400268@muc.edu.cn (T.D.); 21400267@muc.edu.cn (X.L.); 2 Key Laboratory of Ecology and Environment in Minority Areas, Minzu University of China, National Ethnic Affairs Commission, Beijing 100081, China; 3 College of Life and Environmental Sciences, Minzu University of China, Beijing 100081, China; 4 College of Life Sciences, Beijing Normal University, Beijing 100080, China; bingjie@bnu.edu.cn; 5 School of Pharmacy, Hebei University of Chinese Medicine, Shijiazhuang 050200, China

**Keywords:** *Ammopiptanthus nanus*, R2R3-MYB, transcription factor, miR858, anthocyanin

## Abstract

R2R3-MYB transcription factors (TFs) participate in the modulation of plant development, secondary metabolism, and responses to environmental stresses. *Ammopiptanthus nanus*, a leguminous dryland shrub, tolerates a high degree of environmental stress, including drought and low-temperature stress. The systematic identification, structural analysis, evolutionary analysis, and gene profiling of R2R3-MYB TFs under cold and osmotic stress in *A. nanus* were performed. Up to 137 R2R3-MYB TFs were identified and clustered into nine clades, with most *A. nanus* R2R3-MYB members belonging to clade VIII. Tandem and segmental duplication events drove the expansion of the *A. nanus* R2R3-MYB family. Expression profiling revealed that multiple R2R3-MYB genes significantly changed under osmotic and cold stress conditions. MiR858 and miR159 targeted 88 R2R3-MYB genes. AnaMYB87, an miR858-targeted clade VIII R2R3-MYB TF, was up-regulated under both osmotic and cold stress. A transient expression assay in apples showed that the overexpression of AnaMYB87 promoted anthocyanin accumulation. A luciferase reporter assay in tobacco demonstrated that AnaMYB87 positively affected the transactivation of the dihydroflavonol reductase gene, indicating that the miR858-MYB87 module mediates anthocyanin accumulation under osmotic stress by regulating the dihydroflavonol reductase gene in *A. nanus*. This study provides new data to understand the roles of R2R3-MYB in plant stress responses.

## 1. Introduction

Terrestrial plants face various potential environmental threats throughout their life cycles. Abiotic stresses, including water deficit, high salinity, and too-high or too-low temperatures, cause osmotic stress in plant tissues, reduce cell water potential, and cause ion imbalance, thus impairing plant growth, morphogenesis, and photosynthesis [1]. Abnormal weather events have been frequently reported worldwide in recent years, and the occurrence of extreme weather has accelerated desertification [2]. Accentuated environmental stress has reduced the diversity of wild plants and caused damage to crop yields, leading to a food security crisis [3]. Therefore, it is important to investigate the molecular processes and related genes involved in abiotic stress tolerance to protect plant resources and improve crop yields. Most of the molecular mechanisms related to plant stress tolerance have been identified in model plants or major crops [4]. While beneficial, there are still certain limitations, especially the exploration of potentially unique tolerance mechanisms in plants that have inhabited harsh areas for a long time.

*Ammopiptanthus nanus* and *Ammopiptanthus mongolicus*, two rare evergreen broad-leaved shrubs belonging to the genus *Ammopiptanthus* of the family Leguminosae, are mainly distributed in the deserts of Central Asia. *Ammopiptanthus* are relict plants of the Tertiary period that underwent sharp weather changes with the rise of the Tibetan Plateau [5]. Having inhabited harsh environmental conditions for a long time, *Ammopiptanthus* evolved high resistance to environmental stresses, such as water deficit, high salinity, intense radiation, heat, and freezing. *Ammopiptanthus* species are used by local inhabitants as medicines to treat frostbite, chronic rheumatoid arthritis, and disperse petechia. In recent years, multiple bioactive components, such as quinolizidine alkaloids, flavonoids, resveratrol, and polyols, have been identified from *Ammopiptanthus* species [6,7]. Unlike most desert plants, which usually have very small or specialized leaves with a smaller leaf area index (LAI), *Ammopiptanthus* species have typical leaves with a relatively larger LAI, suggesting *Ammopiptanthus* species may have evolved effective mechanisms to survive the harsh environment in the deserts of Middle Asia. *Ammopiptanthus* plants are important for investigating molecular mechanisms associated with abiotic stress responses in trees. Some studies have analyzed the response of *Ammopiptanthus* species to osmotic and cold stress using omics technology, such as mRNA and small RNA sequencing and proteomic analysis [8,9,10,11]. A chromosome-grade genome dataset for *A. nanus* has been released in recent years [12], providing a good starting point for mining genetic factors related to the stress tolerance of *Ammopiptanthus*. 

Myeloblastosis (MYB) transcription factors (TFs) were first identified from the avian myoblastoma virus (derived from the c-MYB proto-oncogene in animal cells) [13]. All MYB TFs identified in different organisms harbor a DNA-binding domain (MYB-DBD) with relatively high conservation at the N-terminus. A number of imperfect tandem repeats (R1, R2, and R3 units) form MYB-DBD, and a single R unit contains three α-helices containing approximately 52 amino acids (aa), which are further folded into a helix-turn-helix (HTH) structure. When MYB TFs recognize a target DNA sequence, the HTH structure enters the major groove to bind to the DNA [14,15]. Plant MYB genes can be further categorized into four classes, namely 4R-MYB, 3R-MYB (R1R2R3-MYB), 2R-MYB (R2R3-MYB), and 1R-MYB (including MYB-related proteins, R3-MYB proteins, and GARP proteins) based on the R unit number [16]. R2R3-MYB TFs mainly contribute to the MYB family scale [16,17]. R2R3-MYB is further classified into different subgroups (SG) in various plant species (usually characterized by sequences outside the MYB domain), and members in the same group have been reported to exhibit comparable biological roles. For example, among the 27 SGs in *Arabidopsis thaliana* R2R3-MYB, S2 has been reported to regulate cold stress tolerance, S9 members modulate epidermal cell differentiation, and S7 is involved in regulating flavonoid metabolism [16,17].

R2R3-MYB TFs are key regulators of secondary metabolism, especially of secondary metabolites biosynthesized in the phenylpropanoid pathway, such as flavonoids, anthocyanins, and stilbenes [18]. Several SGs of R2R3-MYB, including S4, S5, S6, and S7, regulate flavonoid, anthocyanin, and procyanidine synthesis in *Arabidopsis* [17,19]. The R2R3-MYB TF-mediated regulation of anthocyanin accumulation is important for fruit and flower coloration [20]. For example, MrMYB9 was reported to promote anthocyanin accumulation in bayberry fruit by activating several flavonoid pathway-related enzyme genes, such as *CHI*, *F3′H*, and *ANS*, thus deepening the fruit color [21]. *Arabidopsis* MYB75 affects the formation of the MYB-bHLH-WD40 protein complex by interacting with PIF4 and regulating anthocyanin accumulation [22]. FveMYB10 and FveMYB10L, two paralogous R2R3-MYB TFs in diploid strawberries, regulate anthocyanin accumulation in the fruits and petioles, respectively [23]. Additionally, R2R3-MYB TFs regulate both lignin monomer synthesis and polymerization, which are involved in tissue structure support and stress responses [24]. For example, the overexpression of ZmMYB167 in maize promotes the lignification of the secondary wall and increases phenolic content [25]. AtMYB15 participates in defense-response-induced lignification in *Arabidopsis* by affecting lignin monomer biosynthesis-related enzymes [26]. 

R2R3-MYB TFs widely participate in environmental stress responses by regulating multiple molecular mechanisms in plants. For example, PtoMYB142, a poplar R2R3-MYB member, contributes to drought tolerance by regulating wax biosynthesis [27]. The overexpression of *AtMYB44* increases resistance to water deficit and pathogens by regulating stomatal movements [28]. SlMYB15, which is targeted by miR156e-3p, positively regulates ABA-mediated cold tolerance in tomatoes [29]. An R2R3-MYB TF in apples, MdoMYB121, has been reported to maintain the intracellular Na+-K+ balance by regulating water loss and malondialdehyde (MDA) content [30]. The R2R3-MYB TF-mediated regulation of secondary metabolism is also associated with responses to multiple abiotic stresses. *Arabidopsis* AtMYB12 and AtMYB75 participate in drought tolerance by modulating flavonoid and anthocyanin formation [31]. BcCBF2 and BcMYB111 mediate the biosynthesis of flavonoids at low temperatures in Chinese cabbage [32]. The overexpression of *SiMYB16*, a foxtail millet R2R3-MYB gene, promotes flavonoid and lignin accumulation, further enhancing tolerance to salt stress in rice [33].

In recent years, the post-transcriptional regulation of R2R3-MYB by non-coding RNAs has received increasing attention. In model plants such as *Arabidopsis* and rice, miR159/319, miR399, miR828, and miR858 families have been found to target multiple R2R3-MYB genes that are involved in the regulation of plant growth and development, cell cycle, stress response, and secondary metabolism [34,35]. Several miRNA-targeted R2R3-MYB have also been identified in apples, *Salvia miltiorrhiza*, kidney beans, and other plants [36,37,38].

The stress-tolerance-related genes of *Ammopiptanthus* species, including functional genes such as *AnGolS* (codes for the galactoside synthase) [39] and *AnAFP* (codes for the antifreeze protein) [40] and transcription factor genes such as *AmNAC11* [41] and *AnCBF1* [42], have been reported. Nevertheless, few reports have been published on MYB TFs in *Ammopiptanthus* species, and R2R3-MYB TFs have not yet been identified. In this study, we conducted a genome-scale identification of R2R3-MYB TFs in *A. nanus* and analyzed their sequence characteristics, evolution, and expression profiles under stress conditions. We demonstrated that AnaMYB87, an miR858-targeted R2R3-MYB TF, mediates the osmotic-stress-induced accumulation of anthocyanins by activating *A. nanus* dihydroflavonol reductase gene (*AnaDFR*), a gene coding an anthocyanin-synthesis-related enzyme. The present study provides essential evidence for comprehending the roles of plant R2R3-MYB TFs in regulating the adaptation to environmental stress.

## 2. Materials and Methods

### 2.1. Plant Materials and Stress Treatment

The seeds of *A. nanus* were collected from Wuqia County, China. The seeds were surface-sterilized using 70% (*v*/*v*) ethanol for 1 min, followed by bleaching (10%) for 6 min, and then planted in a 30 cm diameter pot containing a 3:1 (*v*/*v*) mixture of vermiculite and perlite. Seedlings were grown in a growth chamber under 120 µmol m^−2^ s^−1^ photosynthetic photon flux density, with a photoperiod cycle of 16 h light and 8 h dark, at approximately 25 °C and 35% relative humidity. The seedlings were watered every 4 days with half-strength Hoagland solution. Eight weeks after germination (the aboveground part of each seedling was approximately 10 cm high with 8–10 leaves, as shown in Figure S1), seedlings with similar growth were randomly divided into nine groups, each containing approximately 50 seedlings. The four groups were directly irrigated with 20% PEG 6000 solution into a perlite/vermiculite mixed matrix for osmotic stress treatment. The other four groups of seedlings were transferred to a 4 °C incubator for the low-temperature stress treatment. The control group continued to grow under the initial conditions. The sampled tissues were snap-frozen in liquid nitrogen and placed at −80 °C for RNA extraction.

### 2.2. Identification of R2R3-MYB Genes

A chromosome-scale genome dataset of *A. nanus* was used as the reference sequence to identify the R2R3-MYB TFs [10]. HMMER 3.0 [43] was used to detect MYB TFs in *A. nanus* with a defined threshold of E < 1 × 10^−5^ using the gene model of the MYB domain (PF00249). All MYB TFs obtained were examined individually using the HMMER web server and the National Center for Biotechnology Information (NCBI) Conserved Domain Search (https://www.ncbi.nlm.nih.gov/Structure/cdd/wrpsb.cgi, accessed on 25 August 2022) to further identify R2R3-MYB TFs. Candidate sequences were also confirmed using NCBI BLASTP (https://blast.ncbi.nlm.nih.gov/Blast.cgi, accessed on 26 August 2022) with an e-value threshold of 1 × 10^−3^ to search for orthologs in *A. thaliana* and soybean. The physicochemical properties of R2R3-MYB TFs were determined using ProtParam (http://web.expasy.org/protparam/, accessed on 2 September 2022) [44]. The Plant-mPLoc (http://www.csbio.sjtu.edu.cn/bioinf/plant-multi/, accessed on 3 September 2022) was used to determine the subcellular localization of the R2R3MYB proteins [45].

### 2.3. Structure Analysis of R2R3-MYB

Chromosomal locations and exon–intron structures were imaged using software TBtools 1.0 (https://github.com/CJ-Chen/TBtools, accessed on 3 September 2022) [46] based on genome annotation. The conserved motifs of R2R3-MYB TFs were detected using MEME (https://meme-suite.org/meme/, accessed on 4 September 2022) [47].

### 2.4. Phylogenetic Analysis of R2R3-MYB

MAFFT v7 (https://mafft.cbrc.jp/alignment/software/, accessed on 4 September 2022) was used to conduct multiple sequence alignments of *A. nanus* and *A. thaliana* R2R3-MYB TFs using the L-INS-I algorithm [48]. ModelFinder [49] was used to choose the substitution model for the phylogenetic analysis according to the Bayesian model. IQ-TREE 2.2.0 (https://www.iqtree.org/, accessed on 4 September 2022) [50] was used to perform maximum likelihood (ML) analysis for inferring phylogenetic trees with 1000 bootstrap replicates, and the phylogenetic tree was imaged using the Interactive Tree of Life (iTOL) v6 (https://itol.embl.de/index.shtml, accessed on 6 September 2022) [51]. The phylogenetic analyses of AnaMYB87 and homolog proteins in *Phaseolus vulgaris*, *Medicago truncatula*, *Trifolium repens*, *Glycine max*, *Lupinus albus*, *Vitis vinifera*, *Petunia hybrid*, *A. thaliana*, and *Oryza sativa* were performed similarly, and sequence alignments were visualized using GeneDoc 2.7.0 (https://github.com/karlnicholas/GeneDoc, accessed on 20 September 2022). 

### 2.5. Gene Duplication Events Analysis

Segmental and tandem duplication events were detected, as described previously [52]. MCScanX (https://github.com/wyp1125/MCScanx, accessed on 21 September 2022) [53] was used to conduct synteny analysis within *A. nanus* genome and synteny analysis of *A. nanus* vs. *A. thaliana* and *A. nanus* vs. *M. truncatula*. KaKs Calculator 2.0 (https://sourceforge.net/projects/kakscalculator2/, accessed on 21 September 2022) [54] was used to calculate the rate of non-synonymous substitution versus synonymous substitution (Ka/Ks) between *A. nanus* R2R3-MYB paralogs.

### 2.6. Identification of Cis-Acting Elements

The New PLACE database (https://www.dna.affrc.go.jp/PLACE/, accessed on 22 September 2022) [55] was used to detect cis-acting elements in the 2000 bp upriver region of the start codon of all R2R3-MYB genes.

### 2.7. Determination of MiRNA-Targeted R2R3-MYB Genes

The sequences of *A. nanus* miRNAs were obtained from a previous report [56]. The psRNATarget v2 software (https://www.zhaolab.org/psRNATarget/, accessed on 22 September 2022) [57] was used to determine the miRNA-target relationship of *A. nanus* R2R3-MYB genes with expectation ≤ 5.0.

### 2.8. Expression Analysis of R2R3-MYB Genes

Transcriptomic data were obtained from GenBank (SRR11089024-SRR11089029 and SRR11087599-SRR11087604). The abundance of each R2R3-MYB gene was estimated using Kallisto quantity [58].

### 2.9. qRT-PCR Analysis

RNA extraction and qRT-PCR analysis of the R2R3-MYB genes, miRNAs, and miRNA precursors were conducted according to two previous reports [56,59]. The *eIF1* gene and an snRNA, U6, were used as references for qRT-PCR analysis of genes and miRNAs, respectively [60]. Three biological replicates were conducted for each group, and three technical replicates were assayed for each biological replicate. The 2^−ΔΔCt^ method was adopted to calculate the relative gene expression level of each gene.

### 2.10. Transactivation Activity Assay

The coding sequence (CDS) of *AnaMYB87* was cloned into the pGBKT7 vector, which resulted in the fusion to the GAL4-BD. Then, the fused vector was introduced into the yeast strain AH109, and the transformed yeast was cultured in the nutrient-deficient media SD/-Trp and SD/-Trp/-His. The empty-vector pGBKT7-transformed yeast cells were used as negative controls, and yeast cells harboring the p53-pGBKT7-fused vector were used as positive controls. Three biological replicates were used for the assay.

### 2.11. Subcellular Localization Analysis

Agrobacterium GV3101 carrying the AnaMYB87-pCAMBIA1303-fused vector suspended in transformation buffer (containing 200 μM acetosyringone, 10 mM MgCl_2_, and 10 mM 2-morpholinoethanesulfonic acid) was used to transform the epidermis of onion sheaths. After 48 h of incubation in MS medium at 28 °C, GFP fluorescence in epidermal cells was visualized using an OLYMPUS Inverted Fluorescence Microscope IX81 (OLYMPUS, Tokyo, Japan) at 488 nm emission wavelength, and 4′,6-diamidino-2-phenylindole (DAPI) was added to the slides and used to stain the nucleus. Three independent biological replicates were used for the assay.

### 2.12. Transient Expression of AnaMYB87 in Apples

The AnaMYB87-pCAMBIA1303-fused vector was transformed into “Fuji” apple fruits through agrobacterium-mediated transformation. Then, injected apple fruits were transferred to a 16 °C incubator with continuous illumination to observe a color change, reflecting the possible accumulation of anthocyanin. Three biological replicates were used for the assay.

### 2.13. Dual-Luciferase Reporter Assay in Arabidopsis

The assay was performed as previously described [61]. Briefly, pre-miR858 of *A. nanus* was fused to pGreen II 62 SK, and the CDS of *AnaMYB87* was fused to pGreen II 0800-LUC. The resulting vectors were co-transformed into *A. thaliana* protoplasts. The luciferase signal was quantitated with the Vazyme Bio-Lite TM Luciferase Assay System Kit (Vazyme, Nanjing, China). The LUC/REN ratio was used to measure luciferase activity. The LUC/REN ratio of the pGreen II 62 SK empty vector with AnaMYB87 was used as a control. Three independent biological replicates were used for the assay.

### 2.14. Luciferase Reporter Assay in Tobacco Leaves

The CDS of *AnaMYB87* was fused to the pRI-GFP vector [62], and the promoter region of *AnaDFR* (ProDFR) was ligated to the pCAMBIA1302-LUC plasmid [63] for expression in tobacco. AnaMYB87-pRI-GFP and ProDFR-pCAMBIA1302-LUC fusion vectors were co-transformed into four-week-old tobacco leaves. After 72 h incubation at 25 °C, luciferase-substrate-sprayed leaves (1 mM D-luciferin with 0.02% Triton X-100) were imaged using a Berthold Night SHADE LB985 imaging system (Berthold, Schwarzwald, Germany) [64]. Three independent experiments were performed.

### 2.15. Determination of Anthocyanin Content

Total anthocyanin was extracted according to a previously described protocol [65]. Briefly, tissue samples were powdered and extracted with 1% HCl-methanol for 6 h. Chloroform and water were then added to the extraction solution, and the absorbance of the extraction solution was measured at 530 and 657 nm. Total anthocyanin content was determined using the following equation: (A_530_ − 0.33 × A_657_)/FW, where FW represents fresh weight. Three independent experiments were performed.

### 2.16. Statistical Methods

For qRT-PCR, the relative gene expression level was calculated using the 2^−ΔΔCt^ method. The least significant difference (LSD) and DunCan Multiple Range test (DMRT), performed using SPSS 24.0 software (IBM, Armonk, NY, USA), were used to conduct multiple comparisons between different time-points. For the dual-luciferase reporter assay and the anthocyanin determination assay, statistical differences between control and experiment groups were evaluated using Student’s *t*-test, with ‘*’ and ‘**’ indicating *p* < 0.05 and *p* < 0.01, respectively. Three independent biological replicates were used for all assays.

## 3. Results

### 3.1. Genome-Wide Identification of R2R3-MYB TFs

**Figure 1:**
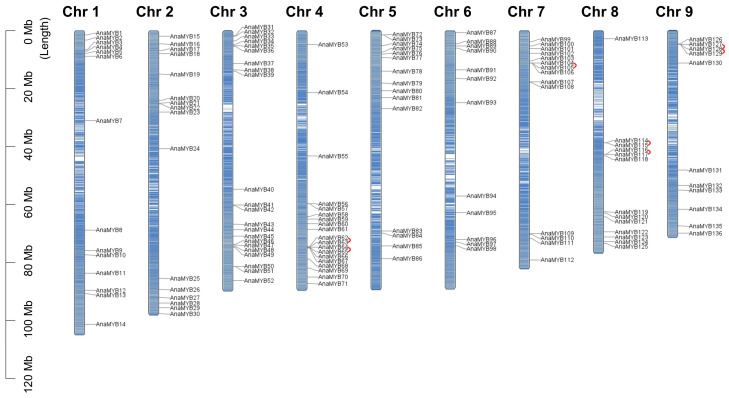
Distribution of R2R3-MYB genes on *A. nanus* chromosomes. Genes linked by curved red lines represent tandem duplication events. The blue line scale inside the chromosomes represents the gene density of corresponding regions.

A total of 137 R2R3-MYB TFs were detected in *A. nanus* (Table S1) and named based on their genomic distribution (Figure 1). The R2R3-MYB TFs of *A. nanus* had an average length of 329 amino acids (aa). AnaMYB60 encoded the largest member, with a molecular weight of 61.99 kilo-Dalton (kDa), whereas AnaMYB26 encoded the smallest member, with a molecular weight of 22.11 kDa. The isoelectric points of the R2R3-MYB TFs ranged from 4.48 (AnaMYB6) to 9.61 (AnaMYB88), and 88 members were acidic (pI < 7.00). The predicted subcellular localization of all R2R3-MYB TFs was in the nucleus. The grand average of hydropathy (GRAVY) scores of all R2R3-MYB TFs were negative, indicating their soluble nature.

*A. nanus* R2R3-MYB genes are located across all nine chromosomes (Figure 1). Of all the R2R3-MYB genes, 22 were distributed on chromosome (Chr) 3, and 11 were distributed on Chr 9. In addition, all *A. nanus* R2R3-MYB genes were located on chromosomes with high gene density, and many genes were located on the chromosome terminus.

### 3.2. Phylogenetic Analysis of R2R3-MYB Family in A. nanus

**Figure 2:**
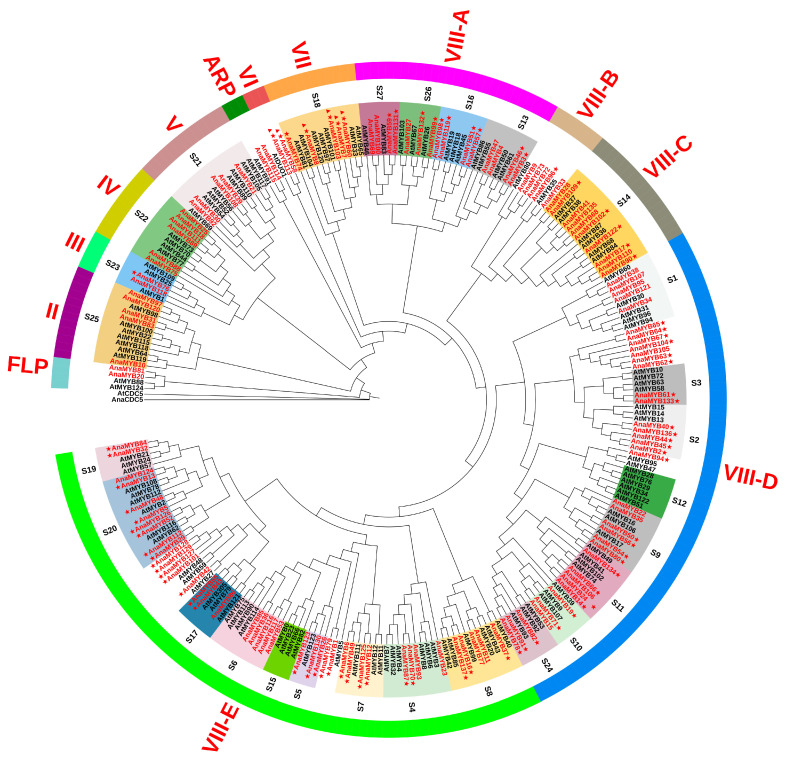
The phylogenetic tree of R2R3-MYB TFs of *A. nanus* and *A. thaliana.* Outer, colored straps represent corresponding clades. Inner, colored blocks represent SGs according to the annotation of *A. thaliana* R2R3-MYB TFs. R2R3-MYB TFs of *A. nanus* were labeled with red font, while R2R3-MYB TFs of *A. thaliana* were labeled with black font. R2R3-MYB TFs of *A. nanus* labeled with triangles or stars represent predicted targets of Ana-miR159 or Ana-miR858.

To investigate the evolutionary status of each R2R3-MYB family TF in *A. nanus*, a maximum likelihood (ML) tree of 137 *A. nanus* R2R3-MYB and 126 *A. thaliana* R2R3-MYB family members was constructed (Figure 2). Two CDC5 proteins were selected for the root trees. *A. nanus* and *A. thaliana* R2R3-MYB TFs were clustered into nine clades. A set of subfamily classifications of terrestrial plant R2R3-MYBs reported by Jiang et al. [66] were used to name these nine clades. Notably, approximately 80% of the R2R3-MYB family members in *A. nanus* were categorized into clade VIII. Clade VIII was further classified into five clusters, namely VIII-A to VIII-E. Subclades VIII-D and VIII-E were significantly more diverse than other subclades of VIII in *A. nanus.*

*A. nanus* R2R3-MYB TFs likely have biological functions similar to those of *A. thaliana* R2R3-MYBs in the same SG; thus, *A. nanus* R2R3-MYB TFs were classified into 25 SGs (Figure 2). Among the 25 SGs, seven groups, including S1, S16, and S23, were associated with stress response regulation; S9, S10, S14, S17, S18, S19, S24, S25, and S26 participated in the morphogenesis and development of tissues and organs; and nine groups, including S3–S8, S21, and S27, were associated with the biosynthesis of secondary metabolites.

### 3.3. Structural Feature of A. nanus R2R3-MYB TFs

**Figure 3:**
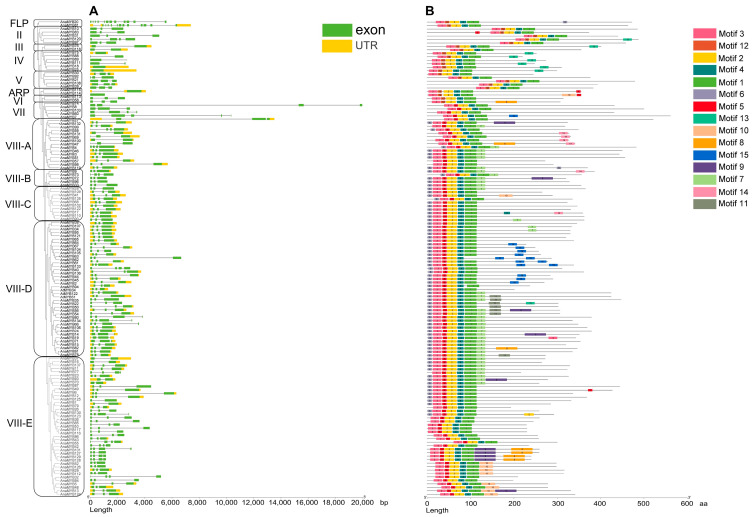
Structural analysis of the R2R3-MYB TFs in *A. nanus*. (**A**) The intron–exon structures of R2R3-MYB genes in *A. nanus*. Black lines represent introns, yellow rectangles represent exons, and green rectangles represent untranslated regions (UTRs). (**B**) The conserved motifs of R2R3-MYB proteins in *A. nanus* predicted using MEME. Colored boxes represent the different motifs.

The majority of *A. nanus* R2R3-MYB genes were shorter than 4 kb, and the shortest R2R3-MYB gene, *AnaMYB114* in clade ARP, was 1070 bp in length. Exon–intron analysis revealed that most R2R3-MYB genes harbored fewer than three introns, and 96 R2R3-MYB genes contained two introns (Figure 3A). However, the FLP members *AnaMYB20* and *AnaMYB81* contained 11 and 12 introns, respectively.

**Figure 4:**
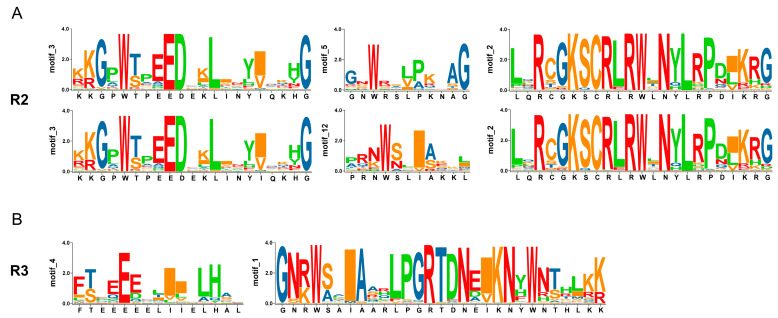
The sequence LOGOs showed motifs overlapping with R2 and R3 units of R2R3-MYB proteins in *A. nanus*. (**A**) Two kinds of sequence LOGOs for R2 unit. (**B**) Sequence LOGOs overlapping with the R3 unit. The relative size of the letters represents their frequency in the sequence. The height of each letter is proportional to the frequency of occurrence of the corresponding base at that position.

Conserved motifs in *A. nanus* R2R3-MYB TFs were predicted, and 15 motifs were located in *A. nanus* R2R3-MYB TFs (Figure 3B and Figure S2). The R2 and R3 units of the R2R3-MYB proteins, containing three and two highly conserved W residues, respectively, were overlapped by five predicted motifs. The R3 units overlapped with motifs 4 and 1, which formed the structure [-F-(X17)-W-(X18)-W-] in all *A. nanus* R2R3-MYB TFs (Figure 4B). Divergence was observed in R2 units in different clades: R2 units of FLP, II, III, IV, V, ARP, and VI overlapped with motifs 3, 12, and 2, which formed the structure [-W-(X19)-W-(X18)-W-], and R2 units of VII and VIII overlapped with motif 3, motif 5, and motif 2, which formed the structure [-W-(X18)-W-(X19)-W-]. Motifs 12 and 5, which contained the second W residue of the R2 unit, covered the divergent region between the two R2 categories (Figure 4A). Several motifs were detected from regions outside the R2R3 domain (Figure 3B and Figure S2), and some of them showed a clade-specific distribution pattern. For example, motif 6 was observed only in type VIII and was the only motif in the upstream region of the R2R3 domain.

### 3.4. Gene Duplication of R2R3-MYB TFs in A. nanus

**Figure 5:**
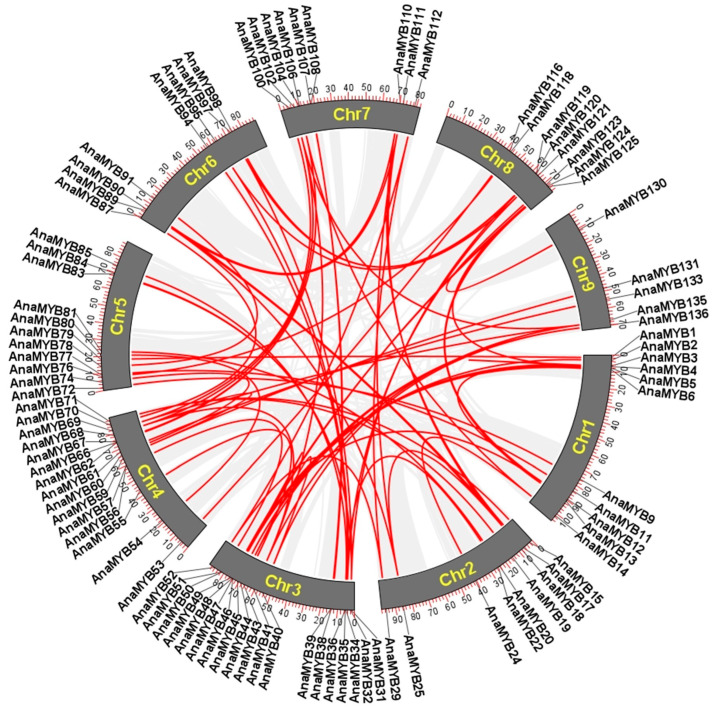
The distribution of segmental-duplicated R2R3-MYB genes on chromosomes of *A. nanus*. Paralogous gene pairs are linked by red curves. Gray lines in the background indicate the collinear blocks.

**Figure 6:**
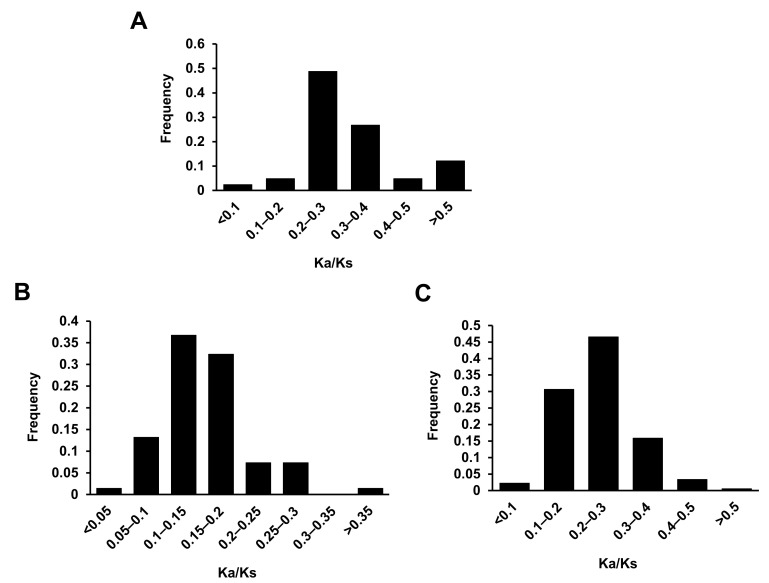
Ka/Ks value distribution of R2R3-MYB genes. (**A**) Ka/Ks value distribution of R2R3-MYB paralog pairs within *A. nanus*. (**B**) Ka/Ks value distribution of R2R3-MYB orthologous pairs between *A. nanus* and *A. thaliana*. (**C**) Ka/Ks value distribution of R2R3-MYB orthologous pairs between *A. nanus* and *M. truncatula*.

Tandem and segmental duplication events were systematically analyzed for R2R3-MYB TFs in *A. nanus*. Six tandem duplication events on four chromosomes of *A. nanus* were identified, involving 13 R2R3-MYB genes (Figure 1). The tandem-duplicated genes belonged to ARP, VIII-D, and VIII-E. Up to 77 potential segmental duplication events were identified, involving 95 R2R3-MYB genes (Figure 5). The segmentally duplicated genes belonged to seven clades, most of which belonged to clade VIII. Moreover, a single R2R3-MYB participated in multiple segmental duplication events in clades II, IV, and VIII. To determine the possible selection pressure on replicated R2R3-MYB genes in *A. nanus*, Ka/Ks was calculated (Figure 6A and Table S2). The Ka/Ks of *A. nanus* paralogous pairs ranged from 0.03 to 0.76, suggesting a purifying selection.

**Figure 7:**
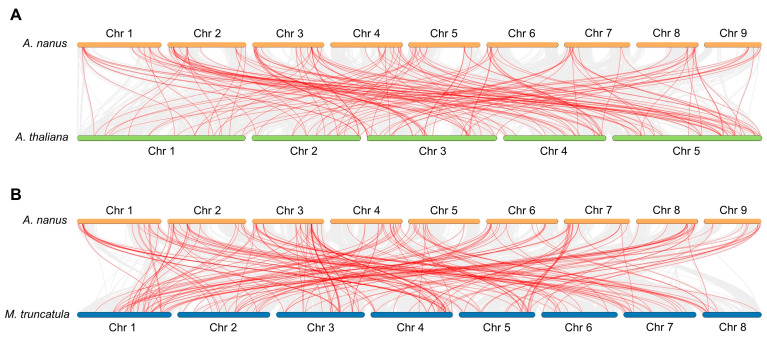
Synteny analysis between *A. nanus* and two plant species revealing orthologous R2R3-MYB gene pairs. (**A**) Orthologous R2R3-MYB gene pairs between *A. nanus* and *A. thaliana*. (**B**) Orthologous R2R3-MYB gene pairs between *A. nanus* and *M. truncatula*. Gray lines in the background indicate the collinear blocks, while the red lines highlight orthologous R2R3-MYB gene pairs.

Synteny analysis between *A. nanus* and two plants, i.e., *A. thaliana* and *M. truncatula*, was performed to determine potential orthologs. In total, 150 R2R3-MYB orthologous pairs between *A. nanus* and *A. thaliana* were identified, including 79 and 86 R2R3-MYB genes, respectively (Figure 7A). In total, 189 orthologous pairs of *A. nanus* and *M. truncatula* were identified (Figure 7B). Up to 73 *A. nanus* R2R3-MYB genes had orthologs in *A. thaliana* and *M. truncatula*, and more than half of these 73 genes belonged to subclades VIII-D and VIII-E. Moreover, the Ka/Ks values of the orthologous pairs of *A. nanus* vs. *A. thaliana* and *A. nanus* vs. *M. truncatula* were calculated. Ka/Ks of *A. nanus* vs. *A. thaliana* orthologous pairs ranged from 0.04 to 0.37 (Figure 6B and Table S3), while those of *A. nanus* vs. *M. truncatula* ranged from 0.08 to 0.50 (Figure 6C and Table S3), suggesting the purifying selection between *A. nanus* and the two plant species.

### 3.5. Cis-Acting Element Analysis of the Promoters of A. nanus R2R3-MYB Genes

To investigate the transcriptional regulation of *A. nanus* R2R3-MYB genes, cis-acting elements in the upstream sequence of the R2R3-MYB CDS were predicted (Table 1). A total of 39 cis-acting elements were found, including 10 associated with the dehydration response, 3 associated with the low-temperature response, 2 related to the pathogen response, and 22 involved in the phytohormone response. Among the cis-acting elements responsive to phytohormones detected in the promoter region of *A. nanus* R2R3-MYB genes, ten were related to gibberellin (GA), four to auxin, three to abscisic acid (ABA), two to cytokinin (CTK), and three to ethylene, jasmonate (JA), and salicylic acid (SA).

Notably, three cis-acting elements were identified in all 137 promoters of *A. nanus* R2R3-MYB family genes, namely MYCCONSENSUAT (ICE1, S000407), ARR1AT (S000454), and WRKY71OS. These three cis-acting elements also had the highest frequency of occurrence among all predicted cis-acting elements in *A. nanus* R2R3-MYB family genes (Table S4).

### 3.6. Identification of A. nanus R2R3-MYB Genes Targeted by MiRNAs

**Figure 8:**
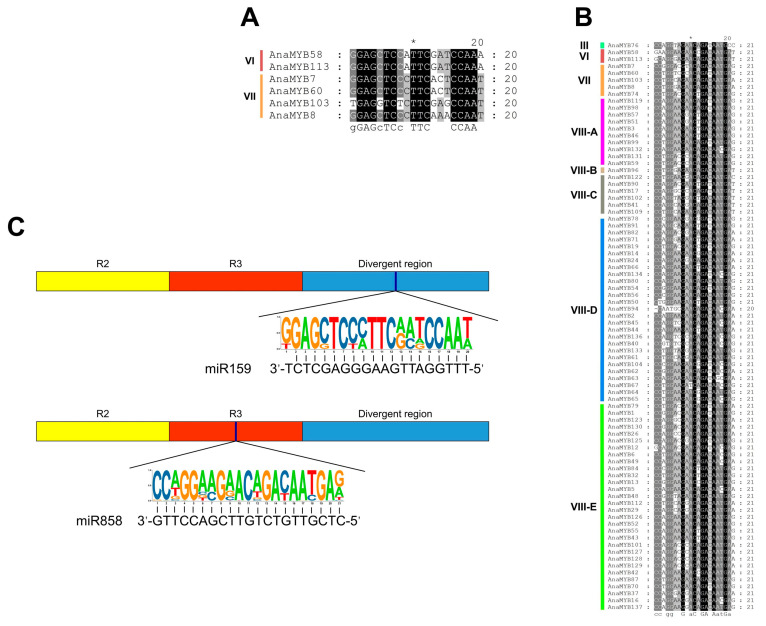
R2R3-MYB genes targeted by miR159 and miR858 in *A. nanus*. (**A**) Sequences of the binding site of Ana-miR159 on R2R3-MYB genes. (**B**) Sequences of the binding site of Ana-miR858 on R2R3-MYB genes. The asterisk (*) indicates the position of the tenth amino acid in the sequence. The background color of amino acids represents the higher (white) to lower (dark) variability of certain site in sequences. (**C**) Locations of binding sites within R2R3-MYB genes.

MiRNAs modulate plant growth, development, and stress responses by negatively affecting their targets, including TF-coding genes. Potential miRNA-targeting R2R3-MYB genes were identified using bioinformatics (Figure 8 and Table S5). A total of 6 Ana-miR159-targeted and 82 Ana-miR858-targeted R2R3-MYB genes were identified, and all predicted R2R3-MYB genes targeted by Ana-miR159 were also targets of Ana-miR858. Two Ana-miR159 targets belonged to subfamily VI, whereas the remaining four belonged to subfamily VII. Ana-miR858-targeted R2R3-MYB genes belonged to III, VI, VII, and VIII (Figure 8A,B).

The binding sites of both Ana-miR858 and Ana-miR159 were located in the CDS of *A. nanus* R2R3-MYB genes. However, the binding sites of Ana-miR858 on R2R3-MYB genes were in the R3 unit, whereas the binding areas of Ana-miR159 were positioned in non-conserved sequences outside the MYB-DBD (Figure 8C). In addition, the binding sites of Ana-miR159 in clade VI were located upstream of the MYB domain, whereas the target sites in clade VII were located downstream of the MYB domain. 

### 3.7. Expression of R2R3-MYB Genes in A. nanus under Cold and Osmotic Stress

Several RNA-Seq datasets of *A. nanus* under stressful conditions were analyzed to investigate the expression profiles of the R2R3-MYB genes (Figure S3). Transcription abundances of *A. nanus* R2R3-MYB genes varied greatly in leaves under normal growth conditions, among which the transcription levels of some R2R3-MYB family members in clades II and VIII were lower, whereas all R2R3-MYB family members of clades FLP and IV were expressed in higher abundance. The induced expression of some R2R3-MYB genes was observed under osmotic and cold stress, e.g., *AnaMYB1*, *AnaMYB15*, and *AnaMYB40* exhibited a 5.41-fold, 4.11-fold, and 21.73-fold increase, respectively, under osmotic stress; *AnaMYB136*, *AnaMYB5*, and *AnaMYB49* increased 4.68, 21.20, and 56.80 times, respectively, under cold treatment; and *AnaMYB53* and *AnaMYB133* were significantly induced by both stresses.

**Figure 9:**
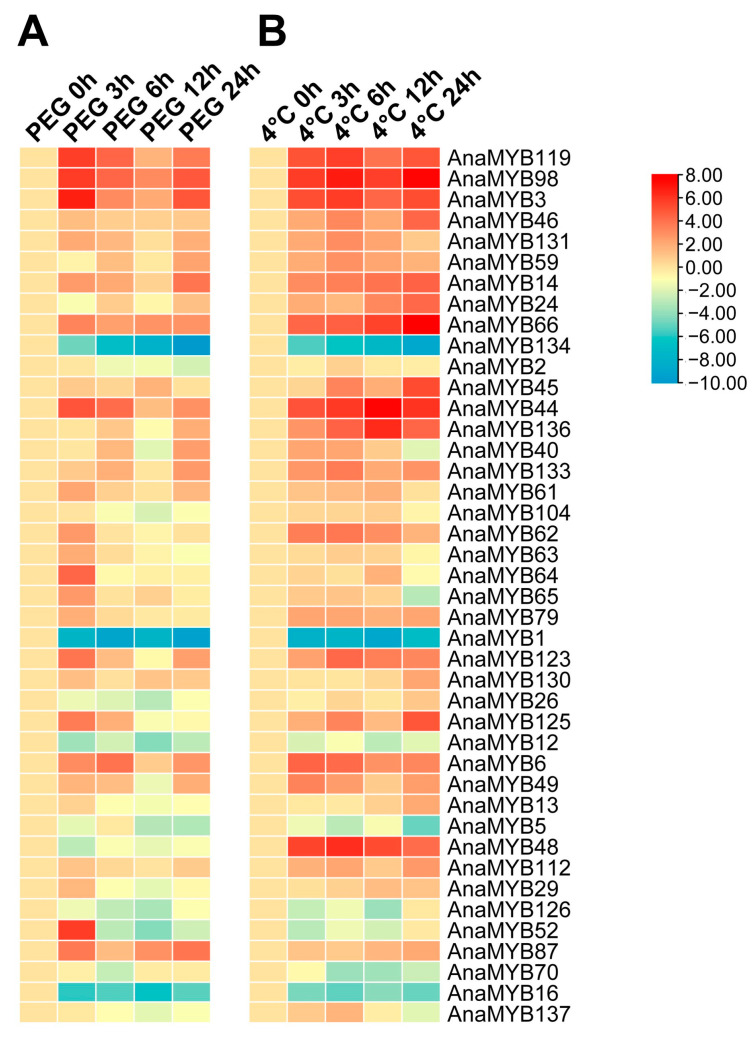
Expression patterns of the *A. nanus* R2R3-MYB genes based on qRT-PCR analysis. (**A**) Expression patterns of R2R3-MYB genes under different durations of osmotic stress. (**B**) Expression patterns of R2R3-MYB genes under different durations of cold stress. The color scale in the heatmap represents higher (red) to lower (blue) expression. Each experiment was performed using three independent biological replicates. The relative gene expression level was calculated using the 2^−ΔΔCt^ method with *eIF1* used as the reference gene and visualized after normalization using logarithm base 2.

To further investigate the dynamic change patterns in the transcription of R2R3-MYB family members under cold and osmotic stress, 42 genes were randomly selected for qRT-PCR analysis after different durations of osmotic and cold treatments (Figure 9). The transcription levels of most selected R2R3-MYB family members were up-regulated more than two times under osmotic or cold stress, except for a few R2R3-MYB genes, such as *AnaMYB1*, *AnaMYB16*, and *AnaMYB134*, which exhibited a significant down-regulation trend. Most selected R2R3-MYB genes showed similar expression patterns under osmotic and cold stress, except for several *A. nanus* R2R3-MYB genes, such as *AnaMYB48*, which exhibited an upward change pattern in cold environments but were down-regulated under osmotic treatment.

### 3.8. Sequence Analysis of AnaMYB87

**Figure 10:**
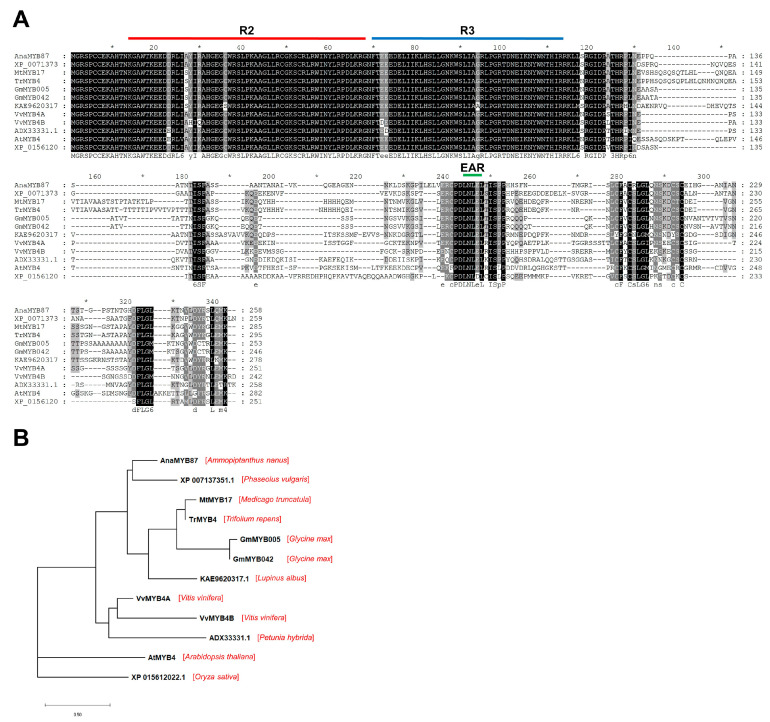
Multiple sequence alignment and phylogenetic analysis of AnaMYB87. (**A**) Multiple sequence alignment of AnaMYB87 and eleven orthologs from nine other plant species showed MYB-DBD (R2 and R3 unit) and EAR motifs. The asterisks (*) indicate the position of amino acid in the sequence from 10th, 30th … to 330th in turn. The background color of amino acids represents the higher (white) to lower (dark) variability of certain site in sequences. (**B**) Phylogenetic analysis of AnaMYB87 and eleven orthologs was performed using IQ-TREE 2.2.0 with 1000 bootstrap replicates.

*AnaMYB87*, an R2R3-MYB gene whose transcription was elevated under both osmotic and cold stress conditions, was selected for further functional investigation. *AnaMYB87* locus is 1171 bp in length and harbors an ORF of 777 bp. Multiple sequence alignment revealed the existence of highly conserved MYB domains that contain R2 and R3 units and an EAR motif (LxLxL pattern) at the C-terminus within AnaMYB87 (Figure 10A). Phylogenetic analysis using AnaMYB87 and its 11 orthologs showed that all MYB family proteins from leguminous species were grouped into a large branch and that AnaMYB87 and its homolog in *P. vulgaris* were clustered into a single clade (Figure 10B).

### 3.9. Transactivation Activity and Subcellular Localization of AnaMYB87

**Figure 11:**
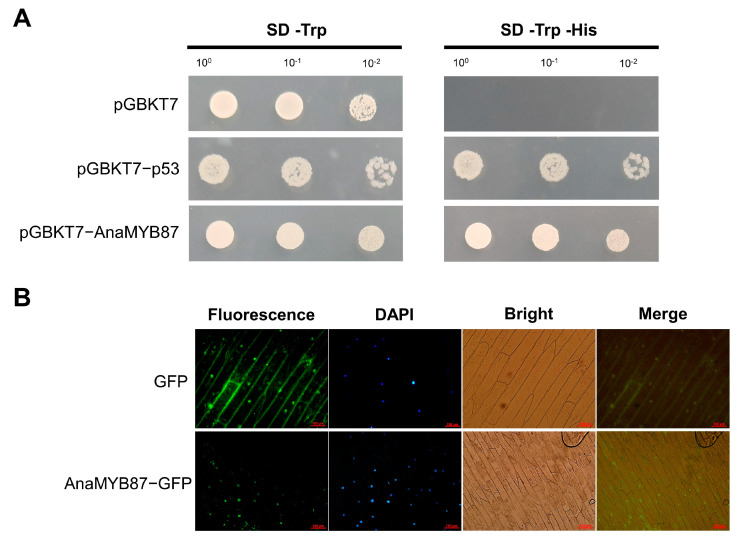
Transactivation activity assay and subcellular localization of AnaMYB87. (**A**) AnaMYB87-transformed AH109 yeast strain survived on SD/-Trp/-His medium, which demonstrated the transactivation activity of AnaMYB87. p53 was used as the positive control. (**B**) Subcellular localization of AnaMYB87 via transient expression in epidermal cells of onion sheath. DAPI was added to the slides to indicate the nucleus. Bars = 100 μm.

Yeast cells containing pGBKT7 or AnaMYB87-pGBKT7 could survive on SD/-Trp medium, while yeast expressing AnaMYB87-pGBKT7 fusion vectors grew well on SD/-Trp/-His plates (Figure 11A), indicating the transactivation activity of AnaMYB87. For subcellular localization analysis, the fusion protein AnaMYB87-GFP was transiently expressed in the epidermal cells of the onion sheath. Fluorescence detection indicated that the GFP signal was distributed in the nucleus and cytoplasm of the onion cells, whereas the fusion protein AnaMYB87-GFP was detected only in the nucleus. The results demonstrated that AnaMYB87 was localized in the nucleus, which is in line with the results of the bioinformatics prediction (Figure 11B).

### 3.10. Transient Ectopic Expression of AnaMYB87 Promoted Anthocyanin Accumulation in Apples

**Figure 12:**
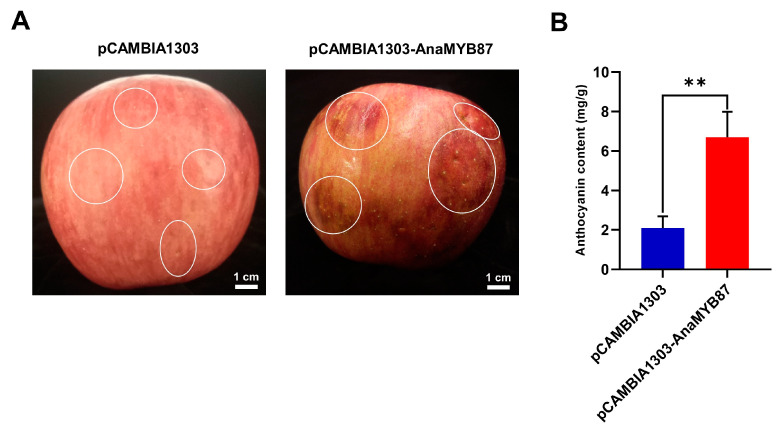
Transient expression of AnaMYB87 promoted anthocyanin accumulation in apples. (**A**) Color change in apple fruit peel after AnaMYB87-fused vectors transformed. Bar = 1 cm. (**B**) The anthocyanin content of transformed peel. Statistical differences were evaluated using Student’s *t*-test with the double asterisks (**) indicating *p* < 0.01.

Evolutionary analysis revealed that AnaMYB87 belongs to the S4 MYB TFs, which participate in the modulation of anthocyanin biosynthesis. Hence, we examined the effect of AnaMYB87 overexpression on anthocyanin accumulation using a transient expression assay. Recombinant vectors with AnaMYB87 inserted were transformed into “Fuji” apple fruits via agrobacterium-mediated transformation. A dark red color was observed in the AnaMYB87-injected regions of the peels, whereas no obvious change was found in the pCAMBIA1303 empty-vector-injected fruits (Figure 12A). The anthocyanin assay further verified that the anthocyanin content of the peel transformed with AnaMYB87 increased by approximately 220% compared to the apple peel expressing the empty vector (Figure 12B). These results indicate that the ectopic expression of AnaMYB87 induces anthocyanin accumulation in apples.

### 3.11. Osmotic-Stress-Induced Anthocyanin Accumulation in A. nanus Leaves

**Figure 13:**
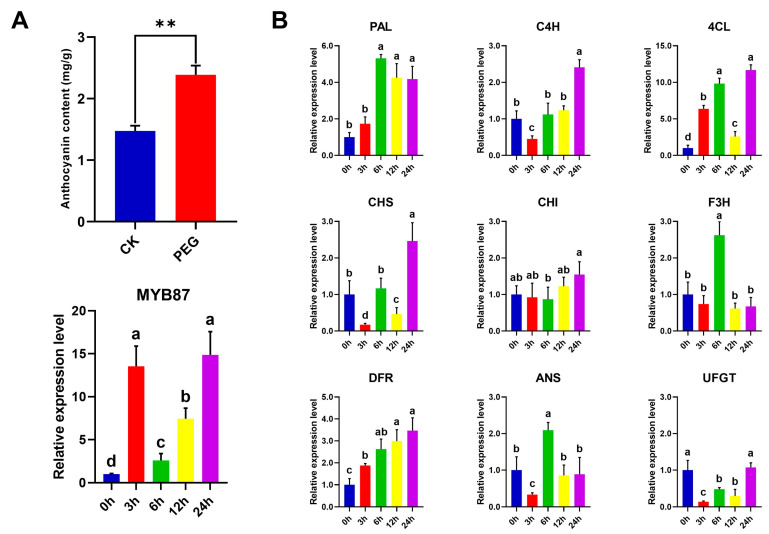
Effects of osmotic stress on anthocyanins and expression of phenylpropanoid pathway-related enzyme genes in *A. nanus*. (**A**) The anthocyanin content of *A. nanus* leaves under normal conditions (CK) and osmotic stress (PEG). Statistic differences were evaluated using Student’s *t*-test with the double asterisks (**) indicating *p* < 0.01. Expression patterns of *AnaMYB87* under osmotic stress based on qRT-PCR analysis. (**B**) Expression patterns of the genes encoding enzymes for anthocyanin biosynthesis in *A. nanus* under osmotic stress based on qRT-PCR analysis. Each experiment was performed using three independent biological replicates. The relative gene expression level was calculated using the 2^−ΔΔCt^ method, with *eIF1* used as the reference gene. The least significant difference (LSD) and DunCan Multiple Range test (DMRT) were used to conduct multiple comparisons. The lowercase letters (a–d) above columns represent the different homogeneous subsets according to multiple comparisons.

Considering that AnaMYB87 was induced by osmotic stress (Figure 9) and that overexpression of AnaMYB87 induced anthocyanin accumulation in apples (Figure 12), it was speculated that osmotic stress may increase the transcription level of AnaMYB87 and cause anthocyanin accumulation in *A. nanus*. Anthocyanin assays revealed that anthocyanin abundance in *A. nanus* leaves increased by 62% under osmotic stress (Figure 13A).

We further analyzed the expression patterns of genes encoding enzymes for anthocyanin biosynthesis in *A. nanus* under osmotic stress (Figure 13B), including *AnaPAL*, *AnaC4H*, and *Ana4CL*, which encode enzymes for precursor biosynthesis in the phenylpropanoid pathway; *AnaCHS*, *AnaCHI*, and *AnaF3H*, which encode key enzymes of the flavonoid branch; and *AnaDFR*, *AnaANS*, and *AnaUFGT*, which encode key enzymes catalyzing anthocyanin biosynthesis. These results indicate that the transcription levels of many functional genes related to anthocyanin synthesis, excluding *AnaCHI* and *AnaUFGT*, were induced by osmotic treatment.

### 3.12. AnaMYB87 Was Targeted by MiR858

**Figure 14:**
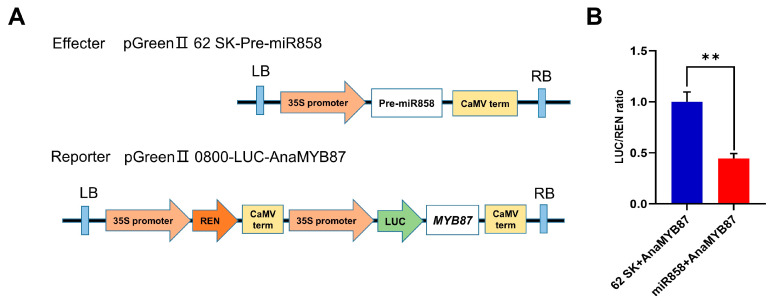
The dual-luciferase reporter assay demonstrated that *AnaMYB87* was targeted by Ana-miR858. (**A**) Fused vectors constructed for dual-luciferase reporter. (**B**) The ratio of LUC/REN in the 62 SK + *AnaMYB87* and miR858 + *AnaMYB87*. Each experiment was performed using three independent biological replicates and statistic differences were evaluated using Student’s *t*-test with the double asterisks (**) indicating *p* < 0.01.

*AnaMYB87* was predicted to be targeted by miR858 using the miRNA target prediction tool psRNATarget, and a dual-luciferase reporter assay using protoplasts of *A. thaliana* leaves was subsequently performed to experimentally verify the target relationship (Figure 14). Fluorescence signal intensity measurements indicated that the luciferase activity of the AnaMYB87-pGreenII 0800-LUC-fused vector decreased with the co-infiltration of miR858 fused to the pGreen II 62 SK vector compared with that of the pGreen II 62 SK empty vector. These results demonstrated that *AnaMYB87* is targeted by Ana-miR858.

### 3.13. Ana-miR858 Was Down-Regulated upon Osmotic Treatment 

**Figure 15:**
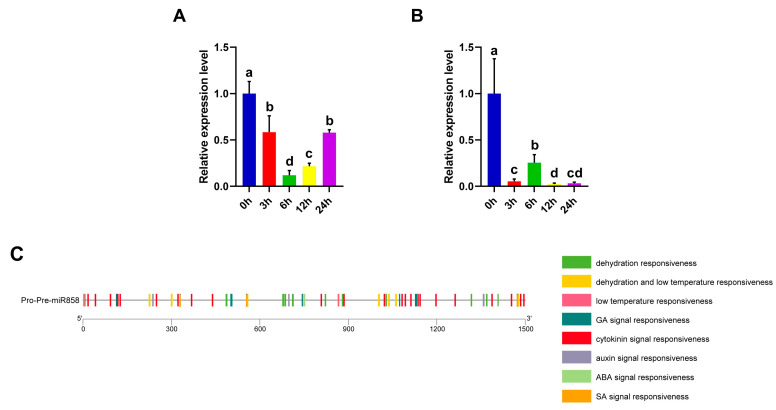
Expression and promoter analysis of Ana-miR858. (**A**) Expression patterns of Ana-miR858 under osmotic stress based on qRT-PCR analysis. An snRNA, U6, was used as the reference. (**B**) Expression patterns of Pre-Ana-miR858 under osmotic stress. Each experiment was performed in three independent biological replicates. The relative gene expression level was calculated using the 2^−ΔΔCt^ method. LSD and DMRT were used to conduct multiple comparisons. The lowercase letters (a–d) above columns represent the different homogeneous subsets according to multiple comparisons. (**C**) Distribution of predicted cis-acting elements involved in abiotic stress response and phytohormones response in the promoter region of Ana-miR858. Different colored blocks represent different types of cis-acting elements.

We further analyzed the transcript levels of Ana-miR858 under osmotic stress conditions using qRT-PCR. Ana-miR858 was down-regulated after osmotic stress (Figure 15A). qRT-PCR revealed that the precursor of Ana-miR858 (Pre-Ana-miR858) exhibited a similar expression pattern under osmotic stress (Figure 15B).

Promoter analysis was performed using the 1.5 kb upstream of Pre-Ana-miR858. The detected cis-acting elements were mainly associated with the response to water deficit and low-temperature stress, and multiple hormone-responsive elements, such as those responsive to GA, cytokinin, auxin, ABA, and SA, were also detected (Figure 15C).

### 3.14. AnaMYB87 Activated the Transcription of AnaDFR

**Figure 16:**
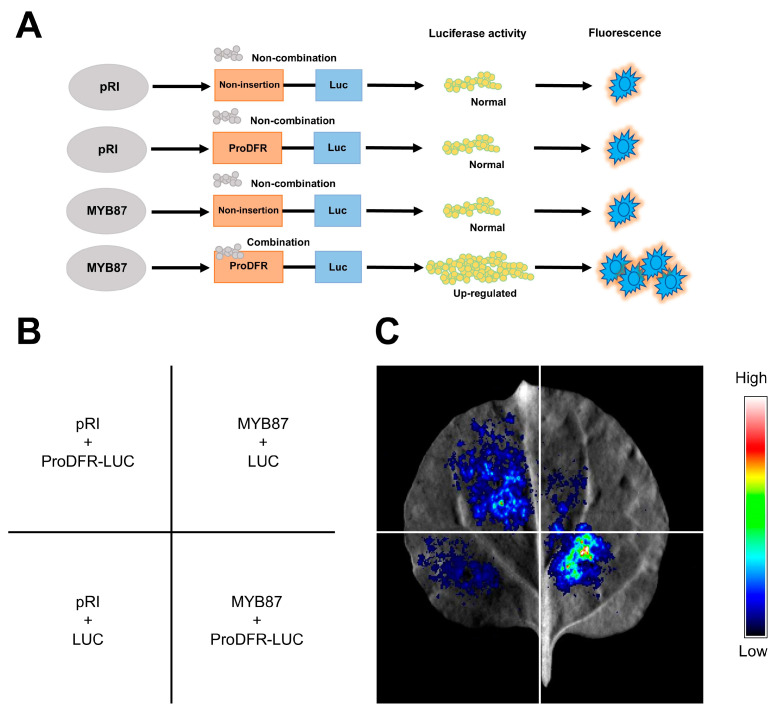
AnaMYB87 positively controls the transcriptional activation of *AnaDFR*. (**A**) The design of luciferase reporter assay. (**B**) Locations of four co-infiltration groups in tobacco leaves. (**C**) Luciferin imaging of tobacco leaves 3 d after co-infiltration. Each group was performed in three independent biological replicates.

Dihydroflavonol 4-reductase (DFR) directly catalyzes anthocyanin synthesis. *Arabidopsis AtDFR* is a direct target of R2R3-MYB TFs [67]. It is speculated that AnaMYB87 may promote the accumulation of anthocyanins in *A. nanus* upon osmotic treatment by activating the transcription of the DFR gene. Thus, we investigated the effect of AnaMYB87 on *AnaDFR* transcription using a well-established transient expression assay in tobacco (Figure 16). Higher luciferase activity was detected when AnaMYB87 was co-infiltrated with the *AnaDFR* promoter than in the other three control groups, demonstrating that AnaMYB87 positively affects the transcriptional activation of *AnaDFR*.

## 4. Discussion

The diversity of MYB TF family structures in plants poses challenges for accurately identifying members of the R2R3-MYB family. The MYB-conserved domain in vertebrates typically contains three or four-and-a-half R units [17,68]. However, the structures of MYB TFs in plant lineages are more diverse, with 4R-MYB, R1R2R3-MYB, R2R3-MYB, and 1R-MYB being discovered unremittingly as subfamilies of MYB TFs [16]. Similar to R2R3-MYB, the DBD of R1R2R3-MYB TFs also included tandem R2 and R3 units [15]. During the identification of R2R3-MYB TFs, certain R1R2R3-MYB TFs might be misclassified as the R2R3-MYB. Here, a few “R2R3-MYB” TFs preliminarily identified in *A. nanus*, such as EVM0034413.1, actually contained three R units. BLAST alignment showed that the *Arabidopsis* homologs of these *A. nanus* MYB TFs were AtMYB3R-3, indicating that these MYB TFs belonged to R1R2R3-MYB. Similarly, we also found that five MYB TFs, such as MtMYB120, were actually R1R2R3-MYB after re-identifying R2R3-MYB family members in *M. truncatula*, as reported in a previous study [69]. 

Some preliminarily identified *A. nanus* “R2R3-MYB” TFs contained two R units, but these two R units are relatively far apart in sequence, reaching 50–200 aa, which differed significantly from the typical R2R3 tandem distribution. The BLAST alignment showed that the homologs of these *A. nanus* proteins were mostly homeodomain (HD) family proteins. Similarly, after re-identifying the R2R3-MYB TFs reported in previous studies, we identified some members of the HD family, such as MtMYB104 in *M. truncatula* [69] and PvMYB16 in *P. vulgaris* [37]. This indicated that MYB domain analysis, phylogenetic analysis, and homologous sequence alignment should be integrated to accurately identify R2R3-MYB TFs in plants.

At present, the R2R3-MYB TFs of multiple plant species have been systematically identified from algae, bryophytes, ferns, and spermatophytes, and classification criteria within the R2R3-MYB of various plants have been proposed based on phylogenetic analysis [16,66]. These classification taxonomies differ greatly, resulting in comparisons of R2R3-MYB between plant species, which are usually limited to a few members of a small branch, making it difficult to compare at the whole-family level. A set of terrestrial plant R2R3-MYB classification systems [66] has recently been used to classify *A. nanus* R2R3-MYB TFs. The classification results for *A. nanus* R2R3-MYB were consistent with those of other plants reported by Jiang et al. [66].

*A. nanus* R2R3-MYB TFs, like their *Arabidopsis* homologs, are involved in regulating the development and multiple metabolic pathways. For example, two clade FLP members, AnaMYB20 and AnaMYB81, were clustered together with *Arabidopsis* AtMYB88 and AtMYB124. AtMYB88 and AtMYB124 were reported to regulate leaf stomatal cell formation and root gravitropism by affecting the G1/S transition in the cell cycle [70,71]. It is speculated that AnaMYB20 and AnaMYB81 are also involved in regulating plant development by affecting the cell cycle. *A. nanus* R2R3-MYB TFs may also participate in secondary wall formation by regulating the synthesis of some metabolites such as lignin. Four members belonging to VIII-A clade, i.e., AnaMYB59, AnaMYB69, AnaMYB100, and AnaMYB131, were clustered with AtMYB46 and AtMYB83 which belong to S27. AtMYB46 and AtMYB83 regulated the transcription of lignin synthesis related enzymes such as hydroxycinnamoyl acyltransferase, p-coumaroyl-CoA-3-hydroxylase, and coumarin-CoA reductase, thereby promoting secondary cell wall formation [72]. We speculate that the above four *A. nanus* R2R3-MYB TFs also participate in the regulation of lignin synthesis. Several *A. nanus* R2R3-MYB members also play roles in cuticular wax biosynthesis. AnaMYB22 and AnaMYB35, which belong to VIII-D clade, were found to cluster with AtMYB16 and AtMYB106, which were reported to cooperate with an AP2 TF, SHN1, to regulate the biosynthesis of cuticular wax in *Arabidopsis* vegetative organs [73]. It is speculated that AnaMYB22 and AnaMYB35 have similar functions in *A. nanus*.

Some studies have indicated that the expansion of the terrestrial plant R2R3-MYB family is associated with whole-genome duplication and tandem replication events [16]. The analysis of gene duplication events in *A. nanus* revealed that both segmental and tandem duplications were involved in boosting the expression of R2R3-MYB family TFs. The tandem-duplicated gene originates from the duplication of the ancestral gene locus in situ, thus forming a gene cluster with a similar structure and function. Thirteen R2R3-MYB genes in *A. nanus* were identified as tandem-duplicated genes, forming six gene clusters distributed across the four chromosomes. Segmental duplication events typically occur in different regions of chromosomes. A total of 95 R2R3-MYB genes, which formed 77 paralogous pairs, were likely associated with segmental duplication events in *A. nanus*, and these genes were distributed on all nine chromosomes. The number of genes involved in segmental duplication was far greater than that involved in tandem duplication, indicating that segmental duplication was the major driving factor for the *A. nanus* R2R3-MYB TF family boost. 

miRNAs are key regulators of post-transcriptional gene expression in various organisms. These non-coding small RNA molecules inhibit translation or cleave target mRNAs, thereby negatively controlling target gene expression. MiRNAs regulate many biological processes in plants, such as growth, development, morphogenesis, and environmental responses [34,74]. In recent years, multiple miRNA families, including miR858, miR828, and miR159/319, have been found to target R2R3-MYB genes in model plants such as *Arabidopsis* and rice, further regulating various biological processes, including growth, stress response, and secondary metabolism [75,76]. In the present study, we systematically identified the R2R3-MYB gene targeted by miRNAs in *A. nanus*. Up to 82 R2R3-MYB genes were identified as targets of miR858, whereas 6 R2R3-MYB genes were identified as targets of both miR159 and miR858. The R2R3-MYB genes and clades targeted by miR858 were more abundant than those targeted by miR159, indicating that miR858 participates in various biological processes by targeting many R2R3-MYB TFs in *A. nanus*. Previous investigations have shown that R2R3-MYB TFs of clades VI and VII targeted by miR159 are mainly involved in regulating the cell cycle, organ development, and morphogenesis [35], while the R2R3-MYB genes targeted by miR858 are mainly associated with the regulation of the phenylpropanoid pathway [76]. Our work revealed that miR858 not only targets R2R3-MYB related to phenylpropanoid metabolism regulation, but also targets R2R3-MYB with multiple functions in *A. nanus*, including the regulation of various biological pathways such as stress response, secondary cell wall formation, and organ development. 

The binding site of miR858 to R2R3-MYB targets is located in the R3 unit, which is a highly conserved region. In contrast, the binding site of miR159 was located outside the MYB-DBD with higher variability. We speculate that miR858 targets highly conserved regions, suggesting that it may has influences on multiple R2R3-MYB genes under osmotic stress in *A. nanus*. Target sites of miR159 are distributed in variable regions of R2R3-MYB genes, indicated that only a few R2R3-MYB members which have the “right sequence” can be regulated by miR159. Previous studies have shown that the MIR159/319 family not only targets the R2R3-MYB gene, but also negatively regulates other gene families, such as the SPL and TCP genes, the SAUR family, and the esterase/lipase/thioesterase family [38]. However, studies on miR858 exclusively showed that it targets R2R3-MYB genes, especially R2R3-MYB members associated with flavonoid and anthocyanin synthesis [76], including AnaMYB87, which was further characterized in the present study.

Anthocyanins are the downstream secondary metabolites of the phenylpropanoid pathway. They have been reported to function in plant tissue coloration, plant development, and environmental stress response, and benefit human health as antioxidants. The biosynthetic pathway of anthocyanins is usually conserved in higher plants and is well-characterized in several plant species [77]. Several environmental stressors induce anthocyanin accumulation, including abnormal temperatures, water deficit stress, and ultraviolet radiation [78].

R2R3-MYB TFs directly regulate multiple enzymes involved in anthocyanin synthesis. The roles of R2R3-MYB in several SGs, including S4, S5, S6, and S7, in anthocyanin synthesis have attracted attention. It is generally accepted that the MBW complex consisting of WDR, bHLH, and R2R3-MYB regulates the biosynthesis of anthocyanins and procyanidines [67]. In vegetative tissues, AtMYB11, AtMYB12, and AtMYB111, which belong to S7, have been reported to positively regulate key enzymes in the flavonoid pathway, including chalcone synthase (CHS), and the products catalyzed by these enzymes are upstream metabolites for anthocyanin biosynthesis. The S6 members of R2R3-MYB, such as AtMYB75 (PAP1) and AtMYB114, have been reported to directly regulate the enzymes involved in anthocyanin synthesis, including DFR, anthocyanin synthase (ANS), and anthocyanin reductase (ANR) [67]. In seeds, the S5 member AtMYB123 (TT2) has been identified as the main regulator of procyanidine (also known as tannin) synthesis [67]. S4 members of the R2R3-MYB TFs are considered inhibitors of the phenylpropanoid pathway. The first reported S4 R2R3-MYB TFs were AmMYB308 and AmMYB330 from *Antirrhinum majus*, which were found to inhibit the synthesis of lignin monomers [79]. S4 members from different species, such as AtMYB4 from *A. thaliana* [80], VvMYB4 from *V. vinifera* [81], and PtrMYB194 from *Populus trichocarpa* [82], were reported to modulate the synthesis of flavonoids, anthocyanins, and lignins. 

In this study, AnaMYB87, an R2R3-MYB TF belonging to the S4 family, was isolated from *A. nanus*. A dual-luciferase assay was performed to confirm the relationship between Ana-miR858 and *AnaMYB87*. Upon osmotic treatment, the transcription of miR858 was down-regulated, the expression of AnaMYB87 was up-regulated, and anthocyanin levels in *A. nanus* leaves increased significantly. This indicates that the regulatory module miR858-AnaMYB87 may be involved in anthocyanin accumulation. The yeast growth assay showed that AnaMYB87 had transcriptional activation activity, and the apple transformation experiment revealed that the overexpression of AnaMYB87 led to an increase in anthocyanin content, indicating that AnaMYB87 promoted the accumulation of anthocyanins.

**Figure 17:**
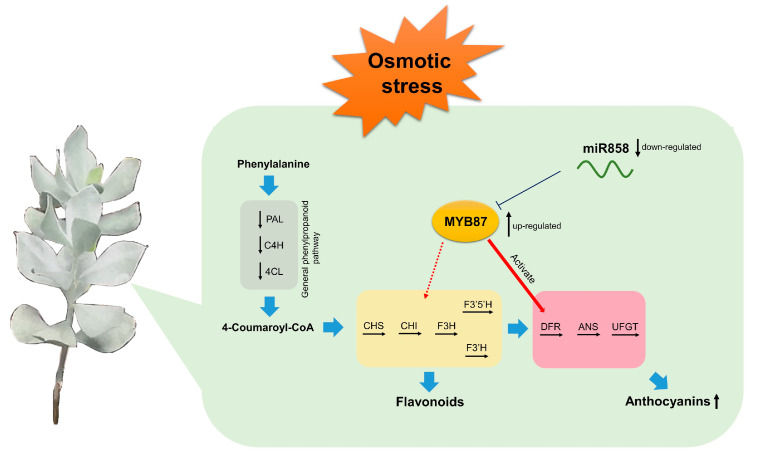
A model for AnaMYB87-mediated regulation of anthocyanins biosynthesis under osmotic stress in *A. nanus*. Under osmotic stress, the expression of miR858 was down-regulated, which weakened post-transcriptional repression on *AnaMYB87*. Up-regulated AnaMYB87 enhanced the transcriptional activation of *AnaDFR* and further promoted the synthesis of anthocyanins in the leaves of *A. nanus*.

A recent study on *Arabidopsis* AtMYB4 (an ortholog of AnaMYB87) showed that AtMYB4 inhibits the expression of CHS and DFR through the MBW complex, thereby affecting the biosynthesis of flavonoids and anthocyanins [80]. Therefore, we explored the possible regulatory relationships between AnaMYB87 and anthocyanin synthesis-related enzymes. A luciferase reporter assay in tobacco revealed that AnaMYB87 interacted with the promoter of *AnaDFR* and activated the transcription of the reporter gene, indicating that AnaMYB87 could activate the expression of *AnaDFR*. As a key enzyme in anthocyanin synthesis, DFR catalyzes the conversion of dihydroflavonol produced by the flavonoid pathway into primary products of the anthocyanin pathway, such as delphinidin, thus promoting the accumulation of anthocyanins. Therefore, we speculate that the miR858-MYB87 module promotes anthocyanin accumulation by modulating the transcription of anthocyanin-synthesis-related enzyme genes such as *AnaDFR*, thus participating in the response of *A. nanus* to osmotic stress (Figure 17).

## 5. Conclusions

In the present study, a total of 137 R2R3-MYB TFs *A. nanus* in were identified and clustered into nine clades. The expansion of the *A. nanus* R2R3-MYB TFs was driven by multiple tandem and segmental duplication events. Some R2R3-MYB genes exhibited significantly changed expression levels under osmotic and cold stress. A total of 88 R2R3-MYB genes in *A. nanus* were targeted by miR858 and miR159. AnaMYB87, induced by drought stress and targeted by miR858, can activate *AnaDFR* in the anthocyanin biosynthesis pathway. The overexpression of AnaMYB87 promoted the anthocyanin accumulation of apples. This study showed that the miR858-MYB87 module mediates anthocyanin accumulation under osmotic stress by regulating the DFR gene in *A. nanus*. 

## Figures and Tables

**Table 1:** The cis-acting elements predicted in the promoter region of *A. nanus* R2R3-MYB genes.

ID	Core Sequence	Cis-Acting Element	Putative Function	FLP	II	III	IV	V	ARP	VI	VII	VIII-A	VIII-B	VIII-C	VIII-D	VIII-E	Total
S000175	CTAACCA	MYBATRD22	Responsive to dehydration	0	1	0	0	1	1	1	0	3	2	2	6	11	28
S000176	CNGTTR	MYBCORE	Responsive to dehydration	2	5	2	6	5	2	2	5	15	5	9	36	41	135
S000177	TAACTG	MYB2AT	Responsive to dehydration	0	1	1	6	2	2	2	3	10	4	1	12	17	61
S000180	GGATA	MYBST1	Responsive to dehydration	2	5	2	6	5	2	2	5	14	5	10	36	39	133
S000408	WAACCA	MYB1AT	Responsive to dehydration	2	5	2	6	5	2	2	5	14	5	9	36	39	132
S000409	YAACKG	MYB2CONSENSUSAT	Responsive to dehydration	2	4	2	6	4	2	2	4	15	5	4	29	32	111
S000413	CATGTG	MYCATERD1	Responsive to dehydration	2	5	1	3	5	2	2	5	11	4	7	23	31	101
S000414	ACGTG	ABRELATERD1	Responsive to dehydration	1	3	2	5	4	2	2	2	15	4	8	30	32	110
S000415	ACGT	ACGTATERD1	Responsive to dehydration	2	5	2	6	5	2	2	3	15	5	9	35	37	128
S000497	RYCGAC	CBFHV	Responsive to dehydration	0	2	1	2	3	0	2	2	6	2	6	14	18	58
S000418	RCCGAC	DRECRTCOREAT	Responsive to dehydration and low temperature	0	2	1	2	1	0	0	2	4	1	4	8	17	42
S000407	CANNTG	MYCCONSENSUSAT	Responsive to dehydration and low temperature	2	5	2	6	5	2	2	5	15	5	10	37	41	137
S000411	GTCGAC	CRTDREHVCBF2	Responsive to low temperature	0	0	0	0	1	0	2	0	0	1	0	2	2	8
S000153	CCGAC	LTRECOREATCOR15	Responsive to low temperature	0	3	2	2	2	0	0	3	10	2	5	19	24	72
S000250	CCGAAA	LTRE-1	Responsive to low temperature	0	2	2	3	2	0	0	3	3	0	1	13	12	41
S000174	CACATG	MYCATRD22	Responsive to ABA	2	5	1	3	5	2	2	4	11	4	7	23	29	98
S000394	ACGTGKC	ACGTABREMOTIFA2OSEM	Responsive to ABA	0	2	2	1	1	0	2	2	0	4	1	9	9	33
S000403	TATCCA	TATCCAOSAMY	Regulation of GA and other hormones	1	3	2	4	2	2	0	2	12	2	9	26	26	91
S000298	TTTTTTCC	Pyrimidine box	Necessity of GA induction	1	1	0	1	1	0	1	3	7	2	2	4	12	35
S000181	TAACAAA	MYBGAHV	Response to GA signal	2	2	2	4	5	1	2	1	7	1	4	20	25	76
S000259	CCTTTT	PYRIMIDINEBOXOSRAMY1A	Response to GA signal	2	5	2	4	5	2	1	4	14	3	9	37	35	123
S000416	TATCCAC	TATCCAC box	Response to GA signal	0	0	1	0	0	1	0	0	5	0	5	3	2	17
S000419	TAACAGA	GARE	Response to GA signal	0	2	1	5	2	1	1	1	7	3	2	9	15	49
S000420	TAACGTA	GARE2OSREP1	Response to GA signal	1	0	0	1	2	0	0	0	0	1	1	8	4	18
S000438	ACGTGTC	GADOWNAT	Response to GA signal	0	2	2	1	0	0	0	1	0	1	1	4	5	17
S000439	TAACAAR	GAREAT	Response to GA signal	2	4	2	4	5	2	2	3	10	2	7	28	32	103
S000447	TGAC	WRKY71OS	Response to GA signal, WRKY binding site	2	5	2	6	5	2	2	5	15	5	10	37	41	137
S000454	NGATT	ARR1AT	Response to cytokinin signaling	2	5	2	6	5	2	2	5	15	5	10	37	41	137
S000491	TATTAG	CPBCSPOR	Response to cytokinin signaling	2	5	0	5	4	1	2	5	13	4	10	34	32	117
S000370	CATATG	CATATGGMSAUR	Response to auxin signals	1	0	1	1	2	1	1	2	12	2	6	20	21	70
S000270	TGTCTC	ARFAT	Response to auxin signals	2	1	1	2	2	2	2	2	11	1	6	23	23	78
S000499	GAGAC	SURE	Response to auxin signals	2	4	1	4	4	2	2	4	15	4	10	35	35	122
S000024	TGACG	ASF-1 binding site	Response to auxin and SA signals	1	4	2	4	3	2	2	4	8	2	4	26	24	86
S000037	AWTTCAAA	ERE	Response to ethylene signal	1	1	1	5	5	1	1	2	11	3	5	24	18	78
S000458	AACGTG	T/G-box	Response to JA signal	0	0	0	2	2	2	1	2	8	3	4	14	12	50
S000292	ACACNNG	DPBFCOREDCDC3	Induced by abscisic acid	2	5	2	4	3	2	1	5	14	5	9	33	39	124
S000390	TTGAC	WBOXATNPR1	Response to SA and other stress signals	2	5	2	6	5	2	2	5	15	5	10	37	40	136
S000391	YTGTCWC	SEBF	Response to pathogenic signals	1	3	0	4	2	2	2	2	10	1	9	23	24	83
S000453	GAAAAA	GT-1 motif	Response to pathogens and high salt stress	1	5	2	5	5	2	2	5	15	5	10	36	36	129

## Data Availability

All data supporting this study are available within the paper and within the Supplementary Materials published online.

## Supplementary Materials

The following supporting information can be downloaded at: https://www.mdpi.com/article/10.3390/biom13121721/s1, Figure S1: The seedlings of *A. nanus* after eight weeks germination; Figure S2: The LOGOs of 15 predicted motifs from the R2R3-MYB TFs in *A. nanus* using MEME. The relative size of the letters represents their frequency in the sequence. The height of each letter is proportional to the frequency of occurrence of the corresponding base at that position; Figure S3: Expression profiles of the R2R3-MYB genes of *A. nanus* calculated from transcriptome datasets. (A) Expression profiles of the R2R3-MYB genes under osmotic stress. (B) Expression profiles of the R2R3-MYB genes under cold stress. The color scale in the heatmap represents higher (red) to lower (yellow) expression. The expression level of each R2R3MYB gene was determined as TPM values from three independent biological replicates of transcriptome datasets and visualized after normalization using logarithm base 2. Table S1: The characterization of the R2R3-MYB genes from the genome of *A. nanus*; Table S2: Ka/Ks of R2R3-MYB paralogous pairs in *A. nanus*; Table S3: Ka/Ks of R2R3-MYB orthologous pairs between *A. nanus* vs. *A. thaliana* and *A. nanus* vs. *M. truncatula*; Table S4: The cis-acting elements detected on promoters of each *A. nanus* R2R3-MYB gene; Table S5: The R2R3-MYB genes targeted by miR159 and miR858 in *A. nanus*; Table S6: The sequences of primers for qRT-PCR analysis.
