# Supplementary figures and images for: The Characterization of R2R3-MYB Genes in Ammopiptanthus nanus Uncovers That the miR858-AnaMYB87 Module Mediates the Accumulation of Anthocyanin under Osmotic Stress
Source: Biomolecules. 2023 Nov 29;13(12):1721. doi: 10.3390/biom13121721 (PMC10741500; doi:10.3390/biom13121721)

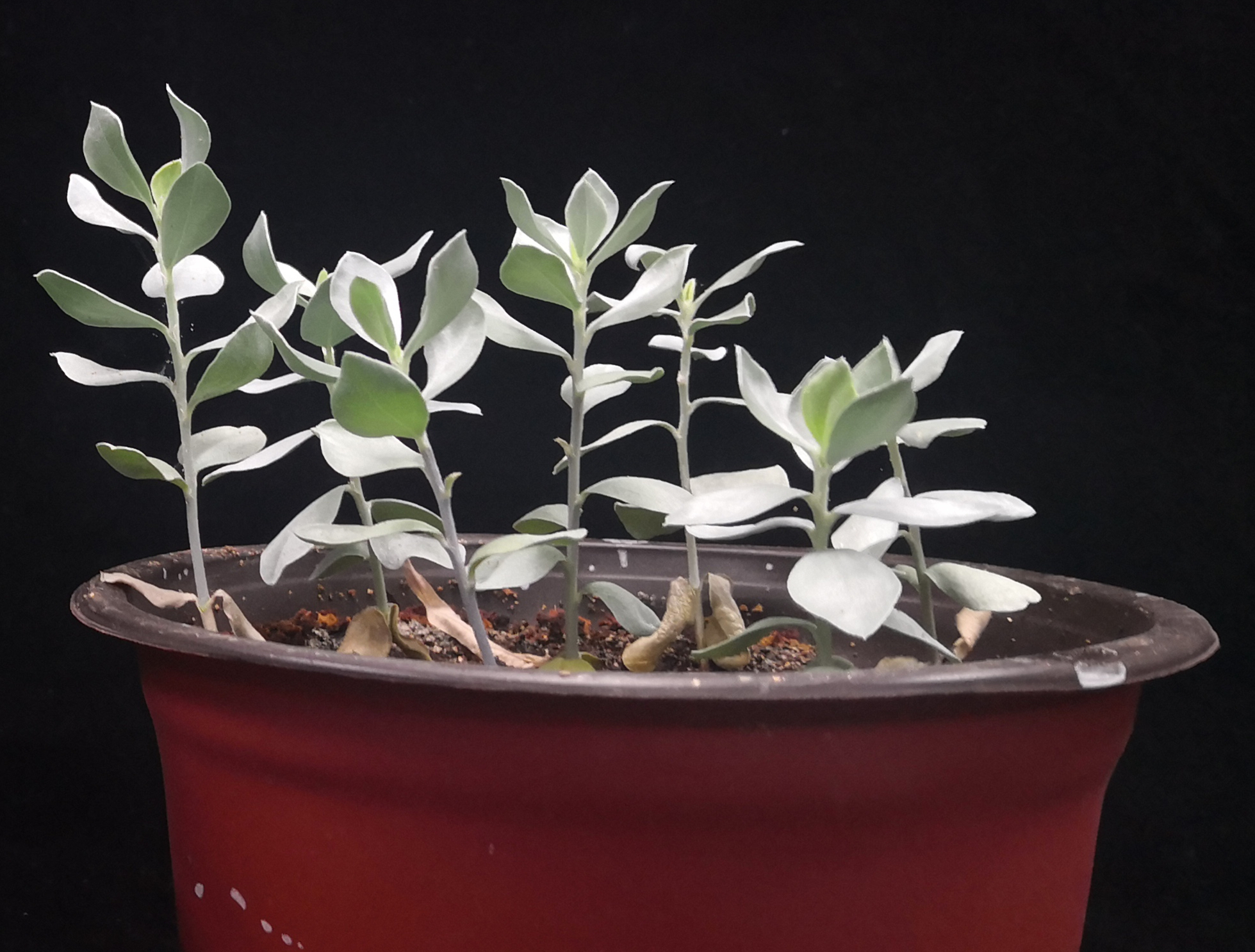

Supplement: Supplementary file 1 [file biomolecules-13-01721-s001.zip › Supplementary material/Fig.S1.jpg]

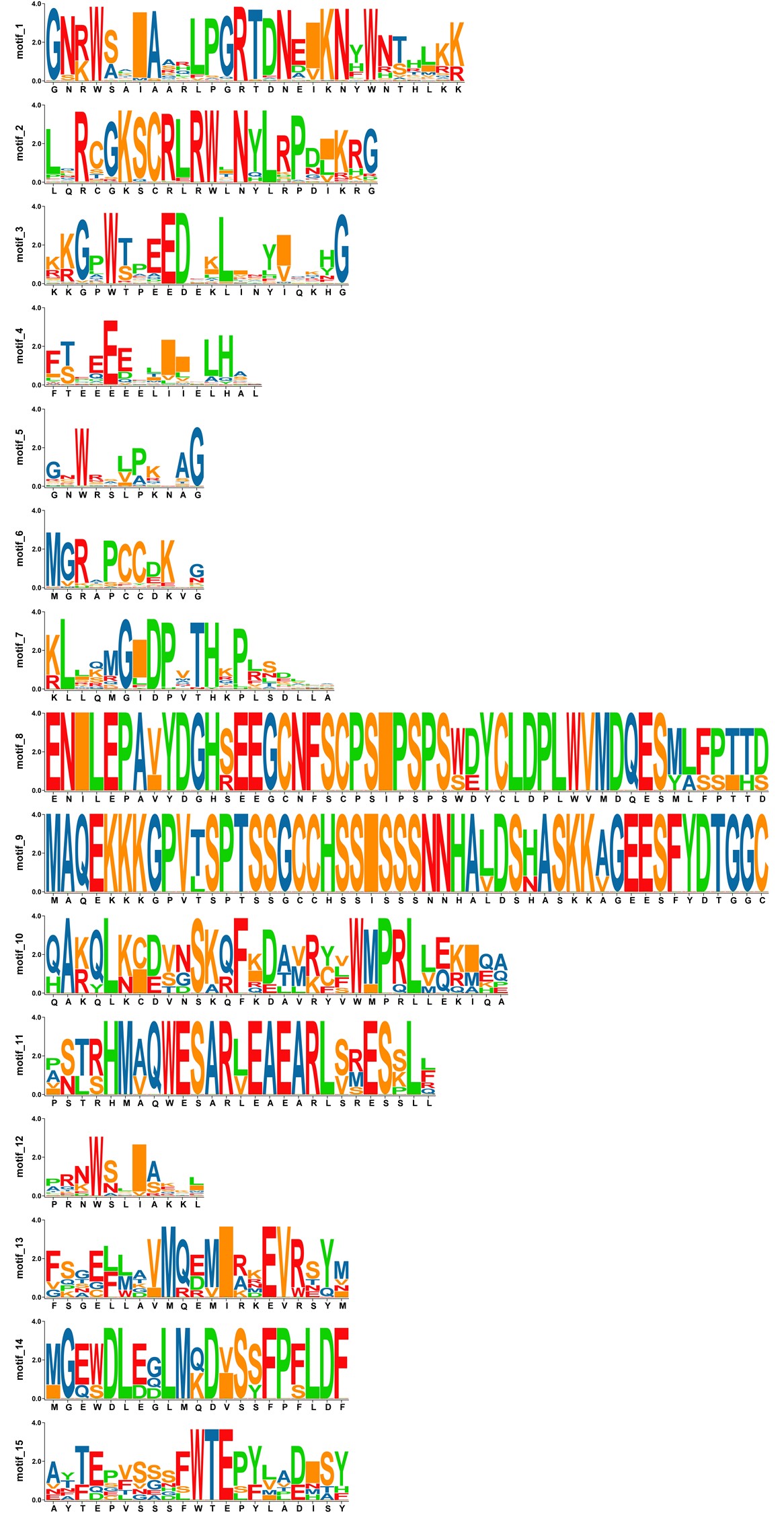

Supplement: Supplementary file 1 [file biomolecules-13-01721-s001.zip › Supplementary material/Fig.S2.jpg]

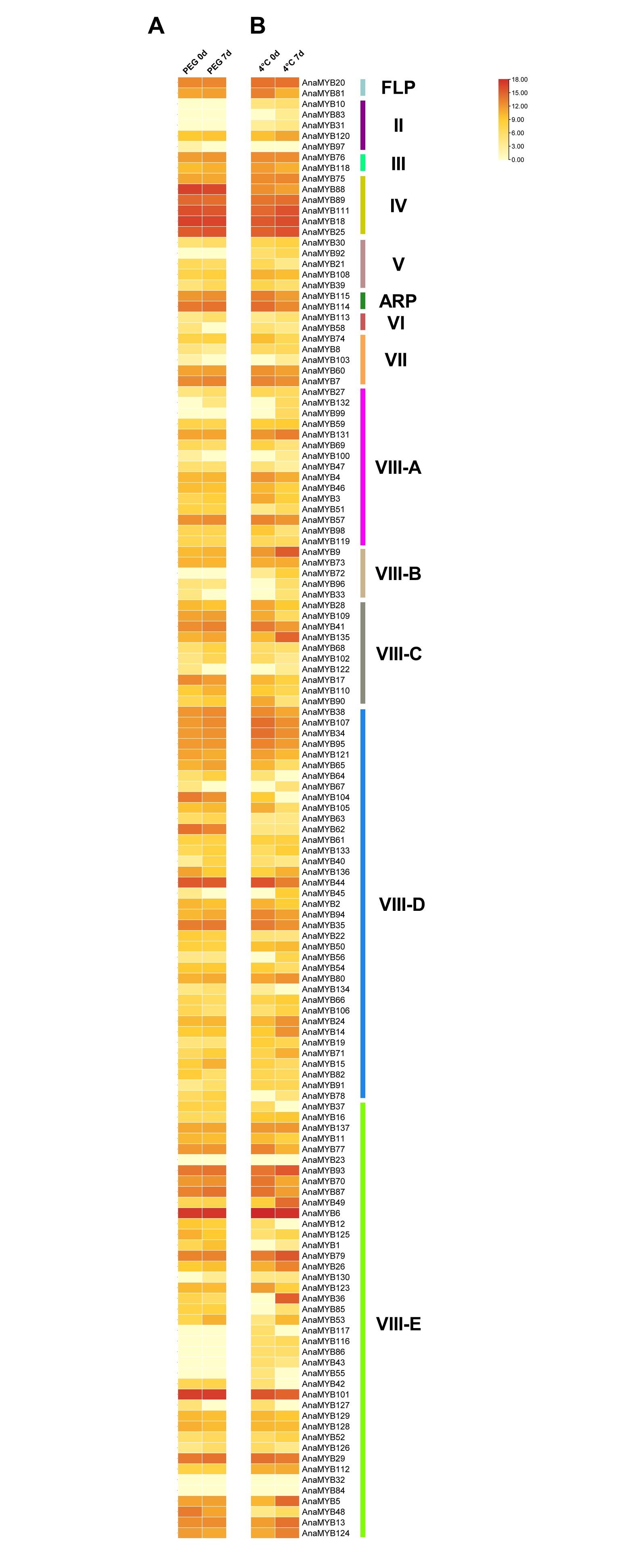

Supplement: Supplementary file 1 [file biomolecules-13-01721-s001.zip › Supplementary material/Fig.S3.jpg]
